# Processing of proprioceptive and vestibular body signals and self-transcendence in Ashtanga yoga practitioners

**DOI:** 10.3389/fnhum.2014.00734

**Published:** 2014-09-18

**Authors:** Francesca Fiori, Nicole David, Salvatore M. Aglioti

**Affiliations:** ^1^Dipartimento di Psicologia, Università degli Studi di RomaRome, Italy; ^2^Laboratiorio di Neuroscienze Sociali, IRCCS Fondazione Santa LuciaRome, Italy; ^3^Department of Neurophysiology and Pathophysiology, University Medical Center Hamburg-EppendorfHamburg, Germany

**Keywords:** rod and frame test, self-transcendence, yoga, field dependency/independency, embodiment

## Abstract

In the rod and frame test (RFT), participants are asked to set a tilted visual linear marker (i.e., a rod), embedded in a square, to the subjective vertical, irrespective of the surrounding frame. People not influenced by the frame tilt are defined as field-independent, while people biased in their rod verticality perception are field-dependent. Performing RFT requires the integration of proprioceptive, vestibular and visual signals with the latter accounting for field-dependency. Studies indicate that motor experts in body-related, balance-improving disciplines tend to be field-independent, i.e., better at verticality perception, suggesting that proprioceptive and vestibular expertise acquired by such exercise may weaken the influence of irrelevant visual signals. What remains unknown is whether the effect of body-related expertise in weighting perceptual information might also be mediated by personality traits, in particular those indexing self-focusing abilities. To explore this issue, we tested field-dependency in a class of body experts, namely yoga practitioners and in non-expert participants. Moreover we explored any link between performance on RFT and self-transcendence (ST), a complex personality construct, which refers to tendency to experience spiritual feelings and ideas. As expected, yoga practitioners (i) were more accurate in assessing the rod's verticality on the RFT, and (ii) expressed significantly higher ST. Interestingly, the performance in these two tests was negatively correlated. More specifically, when asked to provide verticality judgments, highly self-transcendent yoga practitioners were significantly less influenced by a misleading visual context. Our results suggest that being highly self-transcendent may enable yoga practitioners to optimize verticality judgment tasks by relying more on internal (vestibular and proprioceptive) signals coming from their own body, rather than on exteroceptive, visual cues.

## Introduction

The perceived direction of upright, a process referred to as “subjective visual vertical” (SVV), is fundamental for our visual, interpretation of the world. SVV can be assessed by means of the rod and frame test (RFT), developed for the first time by Asch and Witkin (1948). The RFT requires the setting of a visual linear marker (i.e., a rod), embedded in a square luminescent frame, along the gravitational vertical. Importantly, achieving a good performance in the test requires one to ignore the frame. Individuals who are unable to set the rod upright, and instead set it tilted, are also classified as “field-dependent.” Individuals who are able to ignore the misleading context of the frame, setting the rod upright, are classified as “field-independent.” RFT performance depends on the integration of visual with internal bodily signals (e.g., vestibular and proprioceptive), and relies on a multisensory integration process that involves proprioception, vision, vestibular, and postural cues (Zoccolotti et al., 1992, 1993; Golomer et al., 2005; Luyat et al., 2005; Isableu et al., 2008; Lopez et al., 2008). Interestingly a similar process is involved in postural control (Massion, 1994). Changes in any of the above sensory inputs imply that individuals have to redefine the respective contribution of the different sources of information (Ernst and Bülthoff, 2004) for regulating posture and balance.

Self-transcendence (ST) is considered to be a dimension of character based on a synthesis of information about social and cognitive development and descriptions of personality development in humanistic and transpersonal psychology. According to a widely known psychobiological model of personality, inter-individual differences in spiritual feeling and thinking are detected by the Temperament and Character Inventory (TCI) (Cloninger et al., 1994; Gillespie et al., 2003) and cluster into a supposedly stable personality dimension called ST. ST measures the inclination of human beings toward spirituality, and generally refers to identification with everything conceived as a part of a unified whole reflecting the awareness of being an integral part of the universe (Paloutzian and Park, 2005). Highly self-transcendent people are characterized by great awareness of the self and of the environment (Reed, 2008). Cooperativeness (C) is a dimension of character that measures acceptance of other people, while ST captures the degree to which an individual feels that they are a part of nature and the universe at large (Gillespie et al., 2003). C is a dimension of character that defines the maturity of the self as part of a community or society and is linked to concepts like compassion, empathy, and tolerance (Cloninger et al., 1994).

Yoga, in general, involves a series of integrative mind-body exercises involving stretching, balance, bodily alignment, relaxation, meditation, and breathing. Thus, yoga may increase bodily awareness, in particular the perception of one's body in space (Yardi, 2001). We chose to study yoga practitioners because of their special expertise in body awareness. These individuals are characterized by an almost daily practice with enhanced focus on their body position in space (i.e., in vestibular-proprioceptive terms) and also by an overall embodied lifestyle. The focus on sensory experiences is at the core of many movement-based practices such as yoga. Ashtanga yoga (AY) is a branch of Hatha yoga focusing on physical exercise and non-visual experience of the body in space (Benavides and Caballero, 2009; Varambally and Gangadhar, 2012). Like other yoga experts, AY practitioners are involved in meditative practices (David et al., 2014). However, they have to master body representations that are likely involved in ST (Urgesi et al., 2010; Crescentini et al., 2014). One of the aims of body-mind practices is to reach a high level of ST, together with a deep awareness of one's own body and a non-judgmental attitude to life. Hatha yoga is proved to increase body awareness (Mehling et al., 2009), ST and well-being (Macdonald and Friedman, 2009), while mindfulness-oriented meditation (MOM) improves both C and ST (Campanella et al., 2014). It is held that a close relationship exists in the practice of yoga between the achievement of a stable equilibrium through physical exercises (i.e., to correctly execute poses) and an internal balance in a broader sense of living the present in harmony, accepting oneself, and finding peace. Thus, we sought to determine if there is any specific link between two crucial aspects of AY, namely verticality proprioception and balance for execution of yoga positions. Physical exercises and the development of specific skills may “shape” the mind by means of mechanisms of neural plasticity (Froeliger et al., 2012). Moreover, practicing sport typically enhances verticality perception, especially in experts in disciplines requiring a fine postural control (Golomer et al., 2005). Also, awareness of body orientation modulates the perception of the visual vertical (Barra et al., 2012). On the basis of previous literature we expect AY practitioners perform better in verticality estimation and are field independent. One of the aims of body-mind practices is to achieve high levels of ST, together with a deep awareness of one's own body and a non-judgmental attitude to life. C and ST are likely to be the most complex and evolutionary recent aspects of personality. Unlike temperamental variables, that are underpinned by a very wide subcortical and cortical neural network (Cloninger et al., 1994), the character dimensions, particularly ST, may be associated to cortical structures and be prone to the effects of specific environmental inputs (Urgesi et al., 2010; Crescentini et al., 2014). In view of this, we tested whether AY practice influenced the personality traits likely to be more susceptible to plastic changes. What remains unknown is whether personality traits like ST and C are linked to the perceptuo-motor behavior involved in SVV tasks. Here we used RFT to investigate whether expert yoga practitioners are more field-independent than non-experts. We expected that, like other motor experts (Golomer et al., 1999; Vuillerme et al., 2001b; Jola et al., 2011), yoga practitioners should not be very influenced by external visual cues in assessing SVV. We also explored whether differences in ST (probably higher in practitioners) are associated with differences in RFT performance.

## Methods

### Participants

Data collection was performed at the department of Psychology at University of Rome “La Sapienza,” School of Medicine and Psychology, in the period from January to April 2012. A group of 21 AY practitioners (aged 26–53 years, mean 37.14; mean education 18.38 years, range 13–25 years; 13 females) recruited at two AY schools in Rome, and 22 control participants (aged 26–52 years, mean 35.86; mean education 18.59 years, range 11–26 years; 13 females) with no expertise in yoga, or any meditation practice, participated in the study. Participants were matched for age [*t*_(41)_ = 0.59, *p* = 0.561], gender (χ^2^ = 0.036, *p* = 0.85), and education [*t*_(41)_ = −0.178, *p* = 0.860].

AY is a unique style of yoga that focuses on the non-visual experience of body in space. AY is taught in supervised self-practice, with teachers adjusting the student's body if the asanas (postures) are incorrectly maintained, without giving any other visual, or verbal instructions. There are no mirrors in the room; furthermore, the gaze remains focused on defined points on the body or room. Thus, to correctly achieve/maintain yoga asanas, participants must rely on a very good sense of their body in space, and on high skills in interpreting vestibular information coming from the body. Our yoga participants had been practicing for 3 months to 12 years (mean: 4.8 years) and were able to practice from 1st to 4th series of AY levels with 4 meaning the most advanced one (mean: 1.86 series). AY consists of four series/levels characterized by postures of increasing complexity. A practitioner can only advance to the next series if he/she can physically master all postures of that level. AY courses go well-over training weeks and achieving the highest levels may require years. All participants had normal or corrected-to-normal vision, and gave written informed consent prior to participating in the experimental tests. They received information concerning the experimental hypothesis only after completion of the tasks. Participants were paid 7.50 €/h for participation. All procedures were approved by the ethics committee of the IRCCS Santa Lucia Foundation (Rome), and were in accordance with the standards of the 1964 Declaration of Helsinki. All participants were unaware of the purpose of the experiment.

## Rod-and-Frame test

### Apparatus and stimuli

**Figure 1:**
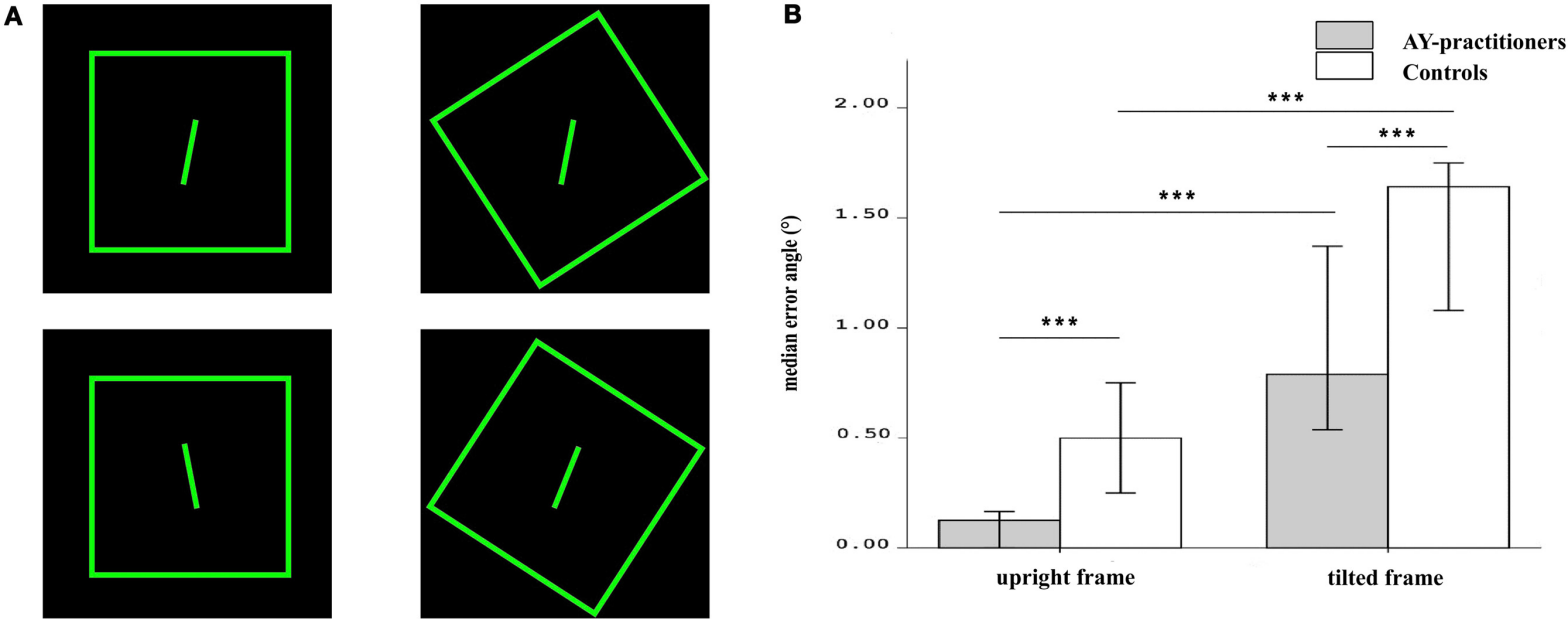
**Example of RFT experimental set-up and results**. **(A)** Participants were seated in front of a large fluorescent frame inside a completely darkened room (left panel). The frame was either tilted or vertical. Inside this frame, approximately at the same level as the eyes, there was a tilted rod, which participants had to verbally set to the vertical (i.e., end angle of the rod 0°). **(B)** Displayed is the median error angle (°), that is the median value of deviation from the gravitational vertical position in the tilted and non-tilted frame condition in both groups. Ashtanga yoga showed significantly smaller deviations of the SVV from the earth vertical when the frame was tilted as compared to novice participants. Furthermore, practitioners and control group, in a with-in comparison, performed better in the upright frame conditions than in the tilted condition. Error bars reflect confidence level at 95%. All tests were two-tailed. Asterisks in the plots indicate significance levels: ^***^p < 0.001.

A standard RFT device square frame was used, previously described in Zoccolotti et al. (1997). Each side of the square frame measured 96 cm, and a single 15 cm long rod was anchored at the center of the frame. Both the frame and the rod were outlined with 1.2 cm-wide fluorescent tape, and were the only visible elements in a completely darkened room. To prevent fading, the apparatus was exposed to light for 30 min before each session. Observers were seated in an erect position at a distance of 160 cm so that the square subtended a visual angle of 34° and the rod a visual angle of 5°. The frame was tilted 33° clockwise (CW), counter-clockwise (CCW), or not tilted (0°), the rod 11° or 22°, CW or CCW. Thus, there were 12 randomly presented conditions, each containing three trials (Takasaki et al., 2012) (see Figure 1A for example). Errors were calculated as deviation from the gravitational vertical position of the rod.

## Assessment of self-transcendence

Participants completed the scale of the Temperament Character Inventory (TCI) assessing ST, and cooperativeness (C), (Cloninger et al., 1994). These two personality traits were selected for their specific relationship with the so-called mind-body practices (i.e., meditation, mindfulness, relaxation, yoga, and tai-chi). Despite several differences, mind-body practices such as mindfulness yoga share meditation goals and do show close relationship with specific personality traits. It has been shown, for example, that MOM (Campanella et al., 2014) after 8-weeks training may increase ST (Campanella et al., 2014). Kemeny et al. (2012) found an increment in empathy and C after 8 weeks of contemplative training. In addition, yoga practice have the potential to increase mindfulness (Shelov et al., 2009), assessed by the Freiburg Mindfulness Inventory (FMI) (Walach et al., 2006). Thus, we sought to determine whether these personality traits are different in AY practitioners with respect to novices.

Individuals high in ST are described as patient, selfless, spiritual, and seem to tolerate ambiguity and uncertainty. The ST scale of the TCI consists of 33 statements, which describe attitudes, opinions, interests or personal feelings and which have to be evaluated as true or false according to the participant's personal opinions. ST includes three subscales: Self-Forgetfulness vs. Self-Consciousness (ST1), Transpersonal Identification vs. Personal Identification (ST2), and Spiritual Acceptance vs. Rational Materialism (ST3). Cooperativeness (C) is assessed with five subscales: Social acceptance vs. intolerance (C1), Empathy vs. social disinterest (C2), Helpfulness vs. unhelpfulness (C3), Compassion vs. revengefulness (C4) Principles vs. self-advantage (C5), and it consists of a total of 42 statements. This scale concerns the degree to which people are generally agreeable, in their relations with others, and how much they identify with and accept others. (see Table 1 for example items).

**Table 1:** **Exemplary Items for each of the sub-scale of the self-transcendence and cooperativeness dimensions as assessed by the temperament and character inventory**.

		**Sub scale**	**Exemplary item**
Self-transcendence	ST1	Spiritual acceptance vs. rational materialism	I believe that miracles happen
	ST2	Self-forgetful vs. self-conscious experience	Often I have unexpected flashes of insight or understanding while relaxing
	ST3	Transpersonal identification vs. self-differentiation	I often feel a strong sense of unity with all the things around me
Cooperativeness	C1	Social acceptance vs. social intolerance	I have no patience with people who don't accept my views
	C2	Empathy vs. social disinterest	I wish other people didn't talk as much as they do
	C3	Helpfulness vs. unhelpfulness	I try to cooperate with others as much as possible
	C4	Compassion vs. revengefulness	Most of the time I quickly forgive anyone who does me wrong
	C5	Pure-hearted conscience vs. self-serving advantage	Principles like fairness and honesty have little role in some aspects of my life

### Procedure and statistical analyses

Participants were blindfolded and led into a dark blue painted room. The concept of verticality was defined using standard examples referring to familiar scenes (e.g., water running from the tap, door frame). Participants had to give verbal instructions to the experimenter in aligning the rod to the gravitational vertical position. No time limit for responses was given. Participants had to keep their eyes closed between trials.

To assess field dep/independency two indexes were used namely the Nyborg and Isaken equation (Frame Effect; Nyborg and Isaksen, 1974) and the Frame Influence. Please note that the Frame Effect is the sum of right-frame tilted trials (CW) divided by the number of right-frame tilted trials, minus the overall mean error in frame tilted condition, calculated by summing up both left-frame tilted (CCW) and right-frame tilted (CW) trials divided by total numbers of trials:

$$\begin{array}{l} \frac{\text{CWtrial1 + CWtrial2 + }\ldots \text{ + CWtrialn}}{\text{nCWtrials}}\\ \;-\frac{\begin{array}{l} \text{CWtrial1 + CWtrial2 +}\ldots \text{+ CWtrialn + CCWtrial1+}\\ \text{CCWtrial2 +}\ldots \text{+ CCWtrialn} \end{array}}{\text{nCCW trials + nCW trials}} \end{array}$$

The Frame Effect index represents the attraction of a perturbing visual field on the subjective vertical. For the purpose of this experiment it was not necessary to know the frame effect direction (CW or CCW). Thus, the frame effect index was used considering absolute values.

A different index of the strategy used in SVV estimation was assessed by subtracting the mean end angle error collapsed against rod and frame upright condition to the mean end angle error collapsed against rod and frame tilted condition (*FR_Tilted_All Rod)-(FR_Non-Tilted_All Rod*). This index (which we called Frame Influence) is independent from the tilt angles and from the participants' ability in estimating verticality, and it may highlight the two different perceptual styles (i.e., field dependency/independency), along a continuum. Higher values indicate that verticality is estimated using mainly a visual strategy. By contrast, low values indicate that verticality is estimated mainly using a proprioceptive or vestibular strategy. The Frame Influence differs from the Nyborg's Frame Effect because it takes into account the errors made by participants in assessing verticality in the upright frame position. The Frame Influence index describes visual context dependency. Frame tilted conditions (CCW and CW) and rod conditions (CCW vs. CW and 11 vs. 22°) were collapsed because the aim of this study was to evaluate the size of the errors in assessing SVV and not the direction of the errors (CCW vs. CW). To test the null-hypothesis that data are normally distributed we used the Shapiro–Wilk test. In the event that data were non-normally distributed, Mann–Whitney and Wilcoxon signed-rank tests were used for between-group and within-group comparisons, respectively. When data were distributed normally, Student *t*-tests were used. Differences in personality traits between AY practitioners and control group have been computed by appropriate between group comparison analysis. To test if any relationship exists between personality traits and bodily processing involved in RFT performance, a correlational analysis was performed. Analyses were conducted using the SPSS software package (version 17.0, SPSS Inc., Chicago, IL, USA). To ascertain if participants are more biased in verticality estimation in CW or CCW frame condition, or when the rod was settled at 11 and 22°, in CW or CCW position, a Friedman ANOVA was performed. If no differences are found, an overall mean across tilted and non-tilted frame conditions can be used.

## Results

### Rod-and-Frame

RTF accuracy analysis was performed by collapsing all rod conditions (11 and 22°, CW and CCW) and then comparing the overall mean error in the tilted-frame vs. the overall mean error in the non-tilted frame conditions. Friedman's test was used before collapsing variables. No differences were found in frame tilted [Friedman test; χ^2^_AY_ (7, *n* = 21) = 7.711, *p* > 0.05; χ^2^_Con_ (7, *n* = 22) = 13.293, *p* > 0.05] and upright [Friedman test; χ^2^_AY_(3, *n* = 21) = 1.691, *p* > 0.05; χ^2^_Con_ (3, *n* = 22) = 3.091, *p* > 0.05] position in both groups. The following variables were then used for the main analysis: FR_Tilted_All Rod represents the mean error of all trials in tilted condition, irrespectively to the rod starting position, and FR_Non-Tilted_ All Rod summarizes all trials in frame upright condition irrespectively to the rod starting position.

To determine differences in accuracy in assessing SVV the overall mean error in the tilted-frame vs. the non-tilted frame conditions were compared in AY practitioners vs. control group. AY practitioners were less biased by the context of a tilted frame in adjusting their SVV (Mdn = 0.79°, IQR = 0.50) compared to controls (Mdn = 1.64°, IQR = 0.72) (Mann–Whitney test; *U* = 88, *p* < 0.001). AY practitioners were also more accurate in judging verticality in the frame non-tilted condition (Mdn = 0.12°, IQR = 0.20) compared to controls (Mdn = 0.50°, IQR = 0.59); [Mann–Whitney test; *U* = 86, *p* < 0.001]. Both groups were more accurate in SVV estimation in the non-tilted condition, (Wilcoxon signed-rank test; *T*_AY_ = 0.00, *p* < 0.001; *T*_Con_ = 1.00, *p* < 0.001), hinting at a similar effect of the presence of the frame (see Figure 1B).

The mean value Ashtanga practitioners Frame Effect was slightly lower (mean = 0.25°, *SD* ± 0.24) than those obtained by controls (mean = 0.37°, *SD* ± 0.30). No significant between group comparison was found [*Frame Effect t*_(41)_ = −1.348, *p* > 0.05]. This indicates that the presence of the frame affected the RFT performance similarly in AY and controls. Frame Effect absolute value, tested against a reference constant (value = 0), turned out to be significant in both groups [*t*_AY(20)_ = 4. 36, *p* < 0.001; *t*_Con(21)_ = 5.76, *p* < 0.001].

In order to analyse the strategy used by participants in assessing SVV, the Frame Influence index was used. AY practitioners (mean = 0.74°, *SD* ± 0.38) and Controls (mean = 1.08°, *SD* ± 0.84) seem to use the same strategy in assessing SVV, *t*_(41)_ = −1.68, *p* > 0.05.

### Observed range in self-transcendence

**Figure 2:**
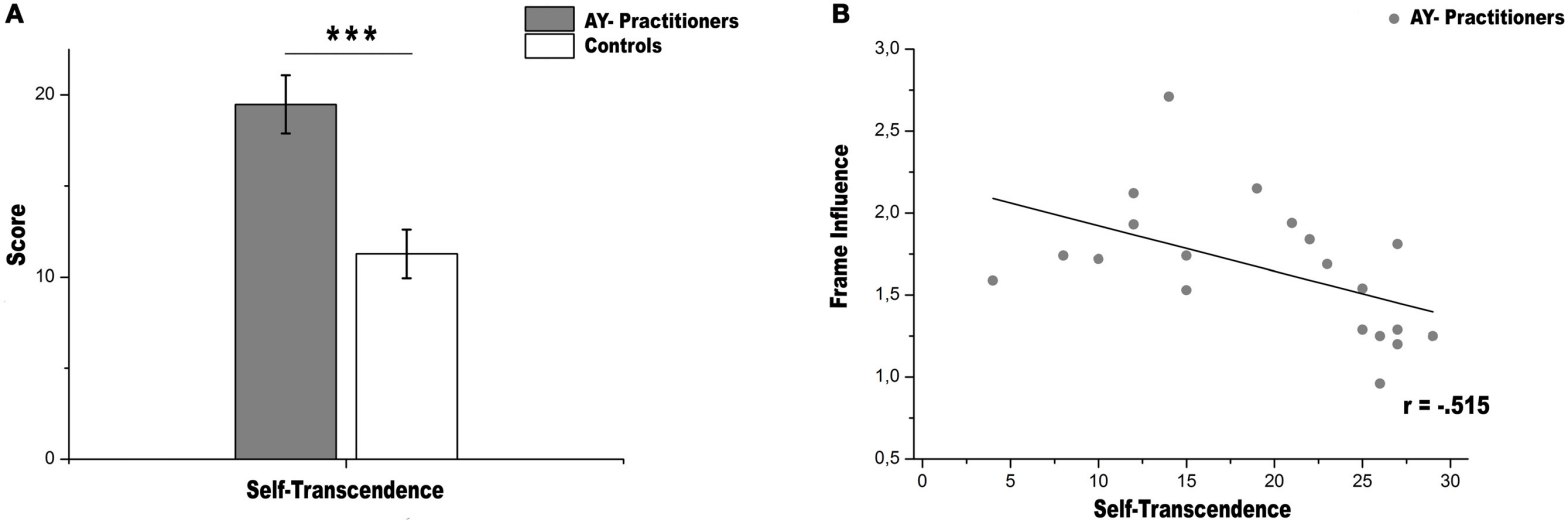
**Personality trait and Rod-and-Frame performance**. **(A)** self-transcendence scores. Ashtanga yoga are significantly more self-transcendent than controls group (error bars reflect s.e.m). Asterisks in the plot indicate significance level, ^***^p < 0.001. **(B)** “Frame Influence” negatively correlates with scores on ST in Ashtanga yoga, proving that the more they use a proprioceptive style strategy in assessing the SVV (small value in “Frame Influence” index), the more they are self-transcendent.

AY practitioners scored significantly higher in ST (mean score = 19.48, range 4–29) compared to control participants (mean score = 11.27, range 2–22); *t*_(41)_ = 3.957, *p* < 0.001 (Figure 2A). AY practitioners scored significantly higher in all ST subscales and since the effect was comparable for the three ST sub-scales, the total ST score was used for the correlation analysis. In the C subscale no statistical difference between groups was found (all *p* > 0.05), therefore relationships between variables were run only for variables statistically different (see Table 2 for all results).

**Table 2:** **Table shows scale range, mean, observed range and differences between groups for each subscale of ST and C scales**.

	**Sub scale**	**Range**	**AY**		**Con**		***t*-value (41)**	***p*-value**
			**Mean**	**Observed range**	**Mean**	**Observed range**		
ST tot	Self-transcendence	0–33	19.48	4–29	11.28	2–22	3.957	0.0003
ST 1	Spiritual acceptance vs. rational materialism	0–13	7.33	1–12	4.56	1–11	2.666	0.011
ST 2	Self-forgetful vs. self-conscious experience	0–11	6.39	1–10	4.37	0–9	2.359	0.023
ST3	Transpersonal identification vs. self-differentiation	0–9	5.76	2–9	2.32	0–6	6.140	0.000003
C tot	Cooperativeness	0–42	35.19	29–40	33.50	18–39	1.362	0.181
C 1	Social acceptance vs. social intolerance	0–8	6.57	1–8	6.81	4–8	−0.564	0.576
C 2	Empathy vs. social disinterest	0–7	6.24	4–7	5.68	2–7	1.675	0.102
C 3	Helpfulness vs. unhelpfulness	0–8	6.86	5–8	6.59	4–8	0.863	0.393
C 4	Compassion vs. revengefulness	0–10	8.29	5–10	7.59	1–10	1.291	0.204
C 5	Pure-hearted conscience vs. self-serving advantage	0–9	7.24	2–9	6.82	4–9	0.923	0.361

### Correlation analysis

Correlation analysis has been run only on Frame Influence and ST score because this index is much more adept to inform about the strategy used by participants to assess verticality. The Frame Effect measures how much the presence of a tilted frame influences the verticality assessment, on the basis of visual distracting cues and not of participants' performance in the upright condition. In our opinion the ability to assess verticality in upright frame condition can be considered as a baseline. This can be important for understanding whether the internal model of verticality is necessarily upright and whether the frame has to be necessarily present in the computing od field dep/independency. Thus, the Frame Influence index describes the ability to remain stable in verticality estimation irrespectively of whether the frame is tilted or not. The Pearson correlation in all participants collapsed together was as follows: *r* = 0.009, *p* = 0.957, *n* = 43. Since the study focused on differences between two groups, the correlation was also performed in the two groups separately. A significant negative Pearson correlation between Frame Influence and ST scores was found in AY practitioners (*r*_AY_ = −0.515, *p* = 0.020, *n* = 20; Figure 2B). Note that one outlier participant was removed from the sample on the basis of the residual analysis. No such correlation was found in the Control group (*r*_*Con*_ = 0.408, *p* = 0.059, *n* = 22). Thus, in AY practitioners, the higher the ST the smaller the influence of the tilted frame.

## Discussion

In this study we investigated the link between the processing of bodily signals assessed by the RFT, AY (a specific type of yoga aiming at increasing body awareness) practicing and dispositional ST. Four main results are reported: (1) AY practitioners performed better than controls in the verticality judgment task in all RFT conditions; (2) both AY and Controls can be considered as field dependent, when using the Frame Effect (Nyborg and Isaksen, 1974); (3) AY practitioners showed higher ST compared to non-yoga practitioners; (4) a negative correlation between “Frame Influence” index and ST scores was found in AY but not in the novice group.

### Processing of sensory inputs concerning body and spatial orienting required for performing RFT and practice of ashtanga yoga

Visual dependence from a given context has been measured in professional dancers who performed RFT with a style more independent from vision with respect to non-dancers (Golomer et al., 1999, 2005). In a similar vein, gymnasts exhibited better postural control than novices (Vuillerme et al., 2001a; Croix et al., 2010), even when the contribution of vision is removed (Vuillerme et al., 2001b). It is also worth noting that in kayak roll athletes who, in structured training sessions, learn specific sub skills (i.e., underwater orientation, paddle movements), greater field-independence (measured with a portable rod and frame apparatus) may parallel fast learning of the training skills. Kayak roll requires cognitive restructuring and strong reliance on kinaesthetic and proprioceptive feedback (Hodgson et al., 2010). AY practitioners typically report the discipline induces an embodied life style, in keeping with the objective need to rely on one's body perception in space for achieving good progress in this discipline. Importantly, any differences between AY and novices in RFT performance cannot be attributed to visual practice, because no visual cues are used in the discipline, no visual feedback is given from mirrors, and the practice is based exclusively on the close connection with the body and the awareness of its position in space.

Thus, we did not find that AY practitioners are more field independent than non-experts. One possible way of interpreting this seeming discrepancy is to consider that our very conservative method to assess field dependency/independency (frame effect = 0) was never used in any previous studies.

While superiority of AY practitioners in the RFT task may not be surprising, no specific conclusion on whether they are more field independent can be drawn.

It is also worth noting that the field-independences as assessed by Nyborg's Frame Effect turned out to significantly differ from 0 in both AY practitioners and novice participants, indicating both groups are equally biased, in evaluating SVV, by the presence of a tilted frame and can be considered field-dependent. In fact, field-independent individuals should not be biased by the frame presence, and their errors (distance in degrees from SVV and earth-gravity) should be near zero, in both frame conditions (tilted or upright). By contrast, field-dependent individuals should make large errors in tilted condition but small errors, if any, in upright frame condition.

The lack of difference in Frame Effect between the two groups is consistent with the assumption that the two groups mainly differ for yoga practice, a physical training designed to increase balance. It is worth noting that no visual cues are used in AY and the practice is based exclusively on the close connection with the body and the awareness of its position in space. Moreover, no visual feedback is given to AY practitioners.

The Frame Influence index describes a perceptive style in assessing the rod in upright position, without taking into account the performance on RTF. Interestingly, the Frame Influence index may allow researchers to virtually divide participants into two sub-groups on a continuum with people who make errors of the same size in tilted and non-tilted frame conditions, and people making large errors in frame-tilted and small ones in non-tilted conditions. People with a high Frame Influence index base their verticality estimation more on the visual system, gaining useful cues in non-tilted condition, but being deceived by the frame in the tilted condition. By contrast, people with a low Frame Influence index adopt a strategy based more on proprioceptive signals without taking the frame angle into account. Thus, individuals who make large errors both in frame tilted and upright condition base their verticality perception on proprioception. By contrast, individuals who provide exact judgment of verticality only in the upright condition, but make large errors in the frame-tilted ones, base their perception of verticality on external visual cues. At any rate, the Frame Influence index may be interpreted as a marker of perception style that gives information about people who determine their verticality perception using more the proprioceptive than the visual system. It is worth emphasizing that the practice of AY usually takes place in places without any mirror and in the absence of visual feedback. Thus, AY practice is based more on proprioception than on visual feedback. No between-group difference in the Frame Influence index was found, indicating that, in spite of the superiority of AY practitioners in performing the RFT task, the two groups did not differ in the strategy used for estimating verticality. This lack of difference may be attributed to the fact that our yoga Asthanga practitioners were mostly beginner-intermediate and no specific analysis on advanced level practitioners could be performed. A possible explanation of this lack of difference may reside in the fact that yoga does increase precision in verticality estimation, but does not induce any change in field-dependency.

### A link between ashtanga yoga and self-transcendence

Our finding shows that yoga practitioners are more self-transcendent than novices. To the best of our knowledge, the study by Büssing et al. (2012) is the only exploring ST related to yoga practice. They adopted a within-subject design, in which Yoga practitioners were enrolled in an intense training for becoming yoga teachers and found showed an increase of ST in yoga practitioners after 6 months of intensive training. These results may suggest that Yoga practice increases ST and specific aspects of practitioners' spirituality, mindfulness and mood. However, ST was higher in yoga practitioners than in the reference control population already before the training. Thus, neither Büssing et al. (2012) study nor our present results can tell apart whether higher ST is the direct consequence of yoga practice or the expression of a tendency to adopt a specific lifestyle.

### Relationship between bodily processing and ST

AY practitioners and the control group did not statistically differ in Frame Influence index. Importantly, however, only in this group there were a relationship between higher ST scores found in individuals with smaller Frame Influence. Thus, highly ST individuals are more in touch with their body, and may be better at analysing information coming from their body. The same correlation was not found in non-yoga practitioners.

Yoga practice is deeply connected with meditative experiences and mindfulness training. There have been reports, for example, of better performance in RTF performance in a group that underwent a 3-months transcendental meditation training compared with a group who did not receive this kind of treatment (Pelletier, 1974). All participants were tested also in an auto kinetic effect and an embedded figure test (EFT; Witkin, 1950) a task designed to assess the concept of field dependence/independence (Witkin and Goodenough, 1981). The meditation group improved their performance in all tasks after 3 months of training. These better performances have been ascribed to an increased field-independence. While this is true for the result obtained in the EFT, participants shifted toward a shorter latency time for the simple figure identification, only a reduction in the error size has been noted in the RFT, and no frame effect was measured. Attention is a critical factor in determining performance in these perceptual tasks. Interestingly one of the most important aims of meditative techniques is to achieve an inward, focused attention. In this context, Pelletier (1974) suggested that these observed differences can be attributed to an alteration in the individual's displacement of attention toward a context-independent cognitive style, due to meditative practice.

It would be interesting to investigate if there is a difference in the “Frame Influence” index between very advanced practitioners (3rd and 4th level), beginners (1st and 2nd level), and novices. Unfortunately in this study it was only possible to collect two practitioners from 3rd level and only one from 4th level.

Our results are partially in conflict with the findings of Hergovich (2003) who showed a relationship between field dependence, measured by EFT, and belief in paranormal phenomena. In Hergovich's study, participants completed several questionnaires assessing belief in paranormal phenomena, while we used the TCI (Cloninger et al., 1994) for assessing ST. A closer inspection of the items revealed that especially the subscale ST-1 shares some elements with the questionnaires administered by Hergovich. For example, in both tests there are items related to phenomena not easily explained by science, alternative medical practices, and near-death experiences. In spite of this possible similarity, it is worth noting that while the questionnaires used by Hergovich (2003) assess beliefs, ST scales assess a specific personality trait. In AY, we found higher field independence and reliance on internal information depending on higher ST scores. An opposite, non-significant tendency, was found in the control group, where high ST scores paralleled field-dependence and reliance on visual cues in SVV tasks. Only the result in healthy controls is in keeping with Hergovich (2003). While we do not have a ready explanation for this partial discrepancy, we note that using different tools for testing field dependency may bring about different results (Arbuthnot, 1972). Thus, although speculatively, we suggest that the difference between the two studies may be explained by the different sensitivity of the tools used. In any case, the different patterns of results in yoga practitioners may be due to a training effect from the embodied experiential practice of AY, that may change reliance on internal signals (i.e., interoceptive, proprioceptive, vestibular). No such learning effect may have occurred in novices. It is worth noting that temporo parietal junction (TPJ), a neural region that is supposed to be an important function in the body's space proprioception (Trousselard et al., 2004; Barra et al., 2012), is also important in body awareness (Bunning and Blanke, 2005; Aglioti and Candidi, 2011), and in integration of signals coming from our own body. It is also interesting that individuals who have experienced out-of-body experiences showed damage to multisensory cortices (Blanke et al., 2004; Lenggenhager et al., 2006; Ionta et al., 2011) centered around TPJ that may also be closely related to the processing of vestibular inputs (Lopez and Blanke, 2011; Lopez et al., 2012). Finally, alterations of TPJ induced by brain lesions (Urgesi et al., 2010), or by inhibitory TMS (Crescentini et al., 2014), induce an increase of ST and of spirituality. The hypothesis that TPJ is involved in both ST (i.e., spirituality) and the perception of the vertical midline has not been clearly tested. However, the question of whether TPJ may be important for performing RTF tasks is currently being investigated at our laboratory.

## Conclusion

We report a relationship between the strategy used to assess verticality and ST only for yoga practitioners. This finding may index some changes in the mechanisms underlying the performance in verticality judgment depending on the personality traits likely influenced by yoga practice itself. More specifically, high levels of ST may guide people to deeper levels of body awareness mediated more by internal (i.e., vestibular or proprioceptive) than external signals. This finding may suggest that individuals who score high in ST have high levels of body awareness and rely more on internal (i.e., vestibular or proprioceptive) than external signals, as proposed by predictive models of interoception (Seth et al., 2011; Seth, 2013). The higher accuracy found in the overall mean, in both frame conditions, may rely on multisensory integrative systems through which optimization of the most reliable information takes place. Moreover, the lack of difference in the strategy used by participants in assessing verticality and in the frame effect index suggests that physical practice can account for accuracy in assessing verticality but not for changes of cognitive style. Yoga practice is a highly embodied discipline that, maybe through an enhancement of ST, allows one to achieve deeper body awareness; this process may be the key for accessing an embodied sense of balance/verticality, and consequently to achieve a more explicit improvement in sensory evaluation.

## Funding

This study was supported by: EU Information and Communication Technologies Grant (VERE project, FP7-ICT-2009-5, Prot. Num. 257695), the Italian Ministry of Health (and RF-2010-2312912) (to Salvatore M. Aglioti), and Deutsche Forschungsgemeinschaft (DA 1358/1-1) and European Union (project “eSMCs”; FP7-ICT-2009-6, #270212) to Nicole David.

### Conflict of interest statement

The authors declare that the research was conducted in the absence of any commercial or financial relationships that could be construed as a potential conflict of interest.
